# Insomnia Among Medical Residents: A Comparative Study Between Medical and Surgical Specialities

**DOI:** 10.1192/j.eurpsy.2026.11676

**Published:** 2026-07-31

**Authors:** G. Ltaief, A. Karmous, O. Ajmi, J. Nakhli

**Affiliations:** Psychiatry Department, Farhat Hached Hospital, Sousse, Tunisia

## Abstract

**Introduction:**

Insomnia is a common problem among medical residents, with studies showing that more than half of them suffer from sleep disorders or insomnia, often linked to stress, anxiety and high workloads.

**Objectives:**

The aim of the study was to assess insomnia among medical residents.

**Methods:**

A cross-sectional study was conducted over three months,from January 2025 to March 2025, using an online survey. Medical residents who were working at Farhat Hached and Sahloul hospitals of Sousse (Tunisia), during the study period were included. Sociodemographic data, medical history and lifestyle habits were collected, supplemented by the Insomnia Severity Index (ISI).

**Results:**

A total of 210 medical residents fully responded to the survey. The mean age was 27.8 years, with 59% being women. The mean number of on-call shifts was 2.45 shifts per month. Residents in medical specialities had a less intense on-call schedule than those in surgical specialities. They had a higher percentage of ‘0 shifts’ (27.3% vs. 0% ; p<0.001), ‘1-2 shifts’ (7.2% vs. 0% ; p<0.001) and ‘3-4 shifts’ (30.9% vs. 15.5%; p<0.001). On the other hand, they had a lower percentage in the ‘5-6 shifts’ (15.8% vs. 42.3%; p<0.001), ‘7-8 shifts’ (16.5% vs 23.9%; p<0.001) and ‘>8 shifts’ (2.2% vs 18.3%; p<0.001).

Residents in surgical specialities had more sleeping problems (66.2% vs. 47.5%; p=0.016). They had also more of subclinical insomnia (54.9% vs. 41.7%; p=0.021) and moderate insomnia (11.3% vs. 4.3%; p=0.017).

**Conclusions:**

Insomnia among medical residents has significant consequences for their mental health and professional performance. Thus, highlighting the need for targeted interventions to improve stress management, sleep hygiene and access to psychological support.

**Disclosure of Interest:**

None Declared

